# Modeling of bud break of Scots pine in northern Finland in 1908–2014

**DOI:** 10.3389/fpls.2015.00104

**Published:** 2015-03-05

**Authors:** Hannu Salminen, Risto Jalkanen

**Affiliations:** Natural Resources Institute Finland (Luke)Rovaniemi, Finland

**Keywords:** *Pinus sylvestris* L., bud burst, phenology, climate, high latitude, tree line

## Abstract

Bud break and height-growth of Scots pine (*Pinus sylvestris* L.) in the northern boreal zone in Lapland, Finland, was followed through the entire growing seasons in the periods 2001–2003 and 2008–2010 in sapling stands in two different locations in northern Finland set some 250 km apart along a latitudinal transect. Field measurements continued at the southern site also in 2011–2013. Air temperature was recorded hourly at the sites. A simple optimization algorithm (GA) was used to adjust parameters of the models predicting the timing of bud break of Scots pine in order to minimize the difference between observed and predicted dates. The models giving the best performance and century-long daily temperatures were used to reconstruct bud-break time series. The temperature observations were recorded for the period 1908–2014 in Sodankylä, which is located in-between the sapling stands in the north–south direction and for the period 1877–2014 in Karasjok, which is in Norway about 145 km north–west from the northernmost stand of this study. On average buds began to extend in the beginning of May in the southernmost stand and in mid-May in the northernmost stands, and the variation between years was in the range of 3 weeks. A simple day-length-triggered (fixed date) model predicted most accurately the date of bud break; root mean square error (RMSE) was 2 and 4 days in the northern and southern site, respectively. The reconstructed bud-break series indicated that based on temperature observations from Sodankylä, growth onset of Scots pine has clearly advanced since the 1960s, though it currently matches that of the early 1920s and early 1950s. The temperature record from Karasjok indicated a similar variation, though there was a weak linear trend advancing bud break by about 3–4 days over a 100-year period.

## INTRODUCTION

Bud break in trees can be seen from different perspectives. The timing of bud break in trees is influenced by both genetic (Beuker, 1994) and climatic factors (Chuine, 2000; Heide, 2003; Linkosalo et al., 2006). Due to the latter, bud break can be linked to changes in spring temperatures, and bud break and other phenophases such as flowering have been used as a bioindicator of climate change. In regard to tree growth, the timing of bud break plays an important role in competition for scarce resources; early growth onset adds length to an otherwise short growing season, although it also exposes the new growth to frost damage often brought on by the varying spring temperatures. In general, bud break has been of interest because it can be visually assessed and is therefore relatively simple to observe.

According to Hänninen (1990), any model for bud break can be presented using three submodels; the rest break, the development of growth competence, and the actual bud development to bursting. The most salient factor in bud-break models is the accumulation of temperature in spring, i.e., forcing. Forcing is either a temperature sum or an equation driven by temperature accumulation. Accumulation is effective after its prerequisite, growth competence, is met. The simpler approach uses day-length to drive growth competence, while the more sophisticated one incorporates the effect of chilling or both (see Chuine, 2000). The development of growth competence and the actual ontogenetic development can be modeled as sequential or simultaneous processes (Hänninen, 1990). The downside of bud-break models is that they are usually driven by daily temperature data, and available continuous daily temperature series from northern Finland are usually relatively recent, typically starting from the early 1960s. In this study, observations of provincial temperature covering a period of over 100 years were applied.

Recent reports on the how the phenology of trees is impacted by climate change are quite unanimous; phenological events have advanced and the growing season has become longer over recent decades (e.g., Chmielewski and Rötzer, 2001; Doi and Katano, 2008; Kaye and Wagner, 2014) or even over the last century (Linkosalo et al., 2009). Empirical data on phenology exist, but the series are usually short, lasting 7–30 years [see a review by Linderholm (2006)] and without reconstruction or incorporating data on flowering or leaf bud burst of broad-leaved tree species. Norway spruce (*Picea abies* (L.) H. Karst.) and Scots pine (*Pinus sylvestris* L.), the two main conifer species in the northern boreal zone in Fennoscandia, have a different strategy in their growth phenology. Susceptible to late frost, Norway spruce delays its flush of the leader bud beyond the beginning of the accumulation of the temperature sum. Therefore the final length of the leader shoot is mainly determined by the current-year temperature. In contrast, the final leader-shoot length in Scots pine is pre-determined by the previous-year summer temperature (Lanner, 1968; Junttila, 1986; Salminen and Jalkanen, 2005). As the temperature of the current season cannot be forecasted, it is rather risky for pine to be determined to grow much in a cold season. To minimize the risk of early frost damage on the leader shoot and bud, pine’s strategy is to flush as quickly as the day length and temperature development alone will allow. Nevertheless, it is very necessary for both species to progress through their phenological phases, including bud break, in a clearly shorter time in northern than more southern latitudes simply to survive in the harsh northern tree-line conditions.

To our knowledge, bud break of Scots pine at high latitudes has not been studied previously using a time-scale of several decades. The aims of this study are (1) to calibrate previously published bud-break models (Hänninen, 1990) with field observations and meteorological data, (2) to test the different types of bud-break models, and (3) to reconstruct a 100-year-long bud break time-series for selected sites using available temperature data in order to establish how reconstructed bud-break dates in northern Finland reflect the supposed climate change.

## MATERIALS AND METHODS

### FIELD OBSERVATIONS

Bud break was followed altogether in five different Scots pine sapling plots representing two different locations in the northern boreal zone in Lapland, Finland, through nine growing seasons in 2001–2003, 2008–2010, and 2011–2013 (**Table 1**). The number of observation years was six in one and nine in the other site, and the same trees were followed for three consecutive years. Thus the empirical material consists of five different tree groups. The height and age of the sample trees in the beginning of the study period were 1.2–3.0 meters and 8–20 years (**Table 1**). For accurate measurement of growth accumulation, a permanent pin was inserted through the main stem of the previous-year shoot. Height growth of the leader shoot of the stem was measured usually at least once a week. The bud break of a plot was defined to take place when more than half of the sample trees had gained at least 1 mm of length and the mean increment of a plot was at least 1% of the total height growth of that season. If those criteria were fulfilled between the once-a-week measurement intervals, which was usually the case, linear interpolation was used to estimate the date for bud break.

**Table 1:** Field observations.

Stand	Plot	Years	Location	Height above see level (m)	Site type	Mean height of sample trees (cm)	Number of sample trees
(1) Laanila, Inari	1	2001–2003	N 68°30′ E 27°28′	215	Dryish heath	169	5
	2	2008–2010	N 68°30′ E 27.28′	215	Dryish heath	178	10^1^

(2) Vanttauskoski, Rovaniemi	1	2001–2003	N 66°22′ E 26°43′	150	Dryish heath	149	15
	2	2008–2010	N 66°22′ E 26°43′	150	Dryish heath	188	5
	3	2011–2013	N 66°22′ E 26°44′	140	Mesic heath	202	10

*^1^Due to mortality, three new sample trees were measured in 2010*.

Air temperature was recorded hourly at the site either during the whole year or throughout the growing season using a Tinytag Ultra data logger (Gemini Data Loggers Ltd.) equipped with an external sensor (range –40 to +125°C and resolution 0.4°C at +25°C). Meteorological measurements taken at 3-h intervals (transformed into mean daily values) were also available from the nearest official climate stations (**Table 2**). The data were calibrated using monthly mean temperature differences between the studied stand and the respective climate station. If onsite measurements were missing, as was the case outside the growing season in some years, respective calibrated values from the nearest official climate station were used.

**Table 2:** Location of the meteorological stations.

Stand	Stand number	Owner	Location	Height above see level (m)	Daily temperature data
Apukka, Rovaniemi, Finland	7502	Finnish Meteorological Institute	N 66°35′ E 26°01′	106	1961–2014
Sodankylã, Finland	7501	Finnish Meteorological Institute	N 67°22′ E 26°39′	179	1908–2014
Ivalo, Inari, Finland	9601	Finnish Meteorological Institute	N 68°40′ E 27°34	123	1958–2014
Karasjok, Norway	97250	Norwegian Meteorological Institute	N 69°47′ E 25°48	155	876–2004
Karasjok, Norway	97251	Norwegian Meteorological Institute	N 69°46′ E 25°50	131	2004–2014

### METHODS

The basic idea of model calibration was simple: a selection of bud-break models were driven by daily temperature data and results were compared to actual observations. First, the daily temperature data for the calibration periods were imported and the bud-break models were implemented in Microsoft Excel (2010). Second, we used an evolutionary solving method (genetic optimization algorithm) add-in included with Excel to select those model parameters that resulted in the smallest root mean square error (RMSE) computed from modeled and observed bud-break dates. Bud break was defined as the date when more than half of sample trees had their leader-shoot bud elongated at least 1 mm and the mean increment of the plot was at least 1% of the total height growth of that season. The following options were set for the evolutionary method: convergence 0.0001, mutation rate 0.1, population size 250, and maximum time without improvement 60 s. Optimization was restricted by setting the allowed bounds for each parameter (**Table 3**). The optimization algorithm screened for the lowest RMSE within the given parameter range. Each model was optimized separately, and the number of parameters depended on the model in question. For example, when fitting the day-length-triggered model, optimization adjusted the parameters of the forcing model as well as the day-length threshold for forcing in order to minimize RMSE. A comparison between the models was based on the outcome of the optimization: the lowest RMSE and the respective parameter values.

**Table 3:** The bounds of the model parameters.

Model	Option	Default	Minimum	Maximum
Forcing	Forcing requirement (FU*_crit_*)^∗^	50	0	250
	*a* in Eq. 1^∗^	28°361	15	40
	*b* in Eq. 1^∗^	0.185	0.01	1.0
	*c* in Eq. 1^∗^	18.431	5	30
Chilling	Chilling requirement (CU*_crit_*)^∗^	20	0	120
	Rest period start date^∗^(fixed)	September 1^st^	September 1^st^	September 1^st^
Day-length	Quiescence period start date	March 15^th^	January 1^st^	May 31^th^

For a detailed parameter description, see Eq. 1 and Hänninen (1990). *default values are taken from Hänninen (1990).

Bud-break models generally include components for rest break, attainment of growth competence and the actual ontogenetic development (Hänninen, 1990, 1995). The first models catch the effect of growth conditions on development from full dormancy to growth competence, and the latter describes how actual forcing eventually results as a bud break. The development from rest to growth competence is usually expressed as a function of accumulated chilling or photoperiod or both. There are various ways in which the development of growth competence can be incorporated in bud-break models (see Hänninen, 1990; Chuine, 2000). We chose to test the four models by Hänninen (1990), which describe how accumulated chilling affects the potential rate of forcing. The models are denoted according to the original article by I_A_, I_B_, II, III, and IV (Hänninen, 1990; **Table 2**). As a comparison, we applied a simple model where a fixed day of year releases the accumulation of forcing units (FUs). That model is denoted by DL (day-length).

Besides modeling the ontogenetic competence, forcing can also be calculated using different types of equations that are usually driven by temperature. In this study, the FU equation based on data by Sarvas (1974) and formulated by Hänninen (1990, Eq. 8) was applied (Eq. 1).

$$m_{frc}\left(t\right)=\{\begin{array}{c} 0\,\;FU\,{day}^{-1},\,\;T(t)\leq 0{}^{\circ}\,\;C\\ \frac{\begin{array}{c} a\cdot FU\,\;{day}^{-1} \end{array}}{1+e^{-b\cdot {}^{\circ}}C^{-1}\cdot \left(T(t)-c{}^{\circ}C\right)}T(t)>0{}^{\circ}C \end{array}\,\;\;\;\;\;\;\;(1)$$

where *m_frc_*(*t*) is the potential rate of forcing in FUs per day, and *T*(*t*) is the prevailing air temperature.

The original parameters (**Table 3**) of this sigmoidal model are based on pooled data for several boreal species (Sarvas, 1974; Hänninen and Kramer, 2007). Therefore, we used the original parameters as starting values and released them to be selected by the optimization algorithm within a predefined range (**Table 3**).

The models giving the best performance in RMSE were used to reconstruct a bud-break date when driven by the longest available daily temperature record measured since 1908 at the Sodankylä meteorological station of the Finnish Meteorological Institute in northern Finland. As a comparison, another time series was constructed based on temperature records from Karasjok, northern Norway available since 1877. The long daily temperature data were imported to the same Excel implementation of the bud-break models as constructed for the calibration phase. The statistical significance of the trend and autocorrelation of the reconstructed bud-break series were assessed with the time-series analysis procedure AUTOREG, which is included with the SAS statistical software (SAS User’s Guide, 2002).

## RESULTS

### BUD-BREAK DATES

The observed bud-break dates during the periods 2001–2003 and 2008–2013 showed large variation in growth onset (**Table 4**). Even during the short 3-year period that each plot was measured, the range of bud-break dates within the same plot covered one week on average, the largest range being 16 days. Trees in Laanila started their growth a few days later than those growing about 300 km south in Vanttauskoski.

**Table 4:** The observed bud-break dates.

Stand	Plot	Years	Bud-break dates
(1) Laanila, Inari	1	2001–2003	17.05.2001, 01.05.2002, and 13.05.2003
	2	2008–2010	26.05.2008, 14.05.2009, and 18.05.2010
(2) Vanttauskoski, Rovaniemi	1	2001–2003	06.05.2001, 30.04.2002, and 09.05.2003
	2	2008–2010	21.05.2008, 10.05.2009, and 13.05.2010
	3	2011–2013	11.05.2011, 12.05.2012, and 14.05.2013

*Bud break was defined as the date when more than half of sample trees had their leader-shoot bud elongated by at least 1 mm and the mean increment of the plot was at least 1% of the total height growth of that season*.

### MODEL CALIBRATION

**FIGURE 1:**
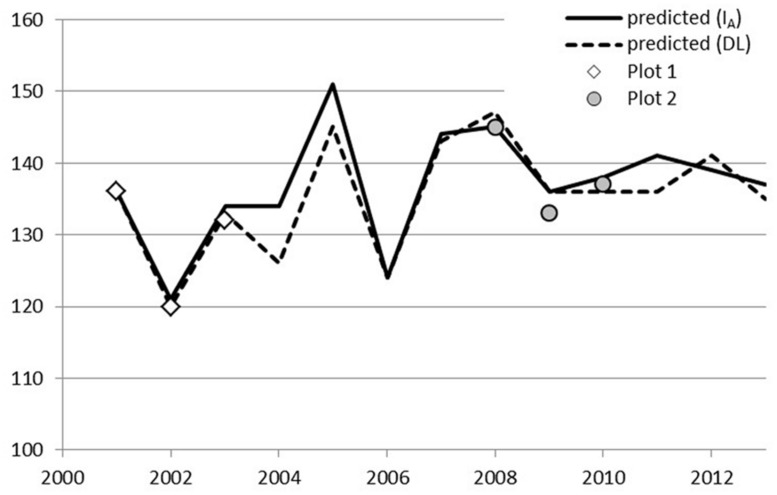
**Predicted and observed bud break in plots 1 and 2 in Laanila, Model I_**A**_ with a chilling component and Model DL with day-length-triggered forcing**.

**FIGURE 2:**
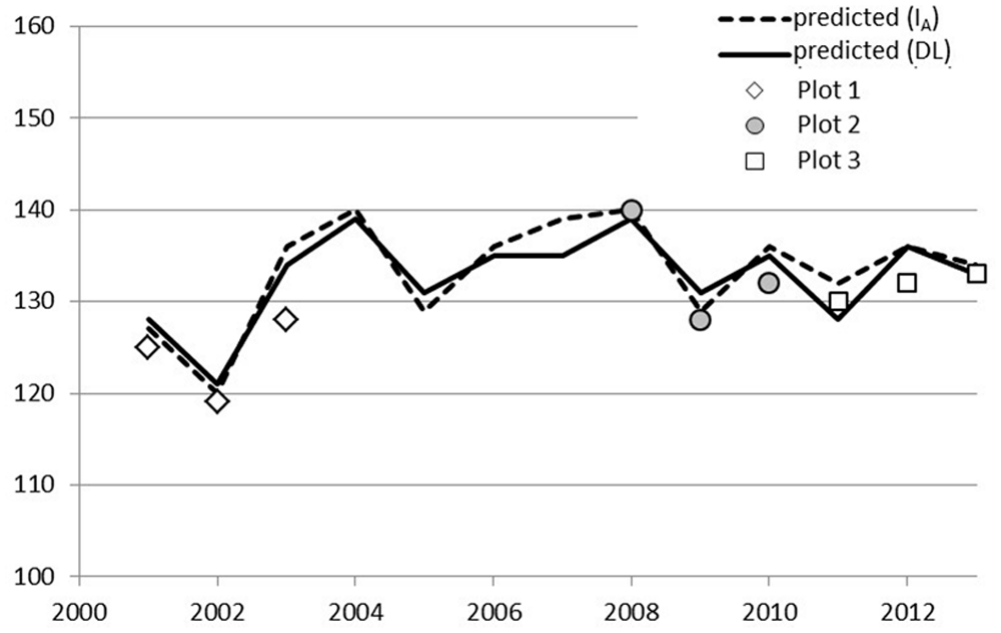
**Predicted and observed bud break in plots 1, 2, and 3 in Vanttauskoski, Model I_**A**_ with a chilling component and Model DL with day-length-triggered forcing**.

A simple day-of-year-triggered model (DL) gave equal performance to the simplest chilling model (Model I_A_, Eq. 2) used in connection with ontogenetic competence. Both of these are sequential; forcing may start after the state of quiescence is attained. Model DL and Model I_A_ resulted in an RMSE of 1.6 and 3.1–3.4 for Stands 1 and 2, respectively, (**Table 5**). The other four models of ontogenetic competence addressed by Hänninen (1990) were inferior to I_A_, so their results are not reported. In Laanila in 2009, bud break took place 3 days before predicted (**Figure 1**). In Vanttauskoski, the years 2003 and 2012 increased the RMSE value the most, partly due to earlier-than-predicted bud break (**Figure 2**).

**Table 5:** Parameters of the bud-break models and their performance (RMSE) after optimization.

Stand (number of observations)	Model	Date or CU-criteria	FU-criteria	Parameters of Eq. 1	RMSE
(1) Laanila, Inari (*n* = 6)	DL	March 30^th^	124.2	*a* = 19.8; *b* = 0.73; *c* = 6.1	1.58
	I_A_	15.4	127.9	*a* = 31.9; *b* = 0.53; *c* = 7.8	1.58
(2) Vanttauskoski, Rovaniemi (*n* = 9)	DL	March 7^th^	196.2	*a* = 21.5; *b* = 0.47; *c* = 6.0	3.12
	I_A_	21.7	112.4	*a* = 15.8; *b* = 0.95; *c* = 6.2	3.45

$$M_{chl}\left(t\right)=\{\begin{array}{cc} 0\,\;CU\,day^{-1}, & T(t)\leq -3.4{}^{\circ}C\\ 0.159\,\;CU\,day^{-1}{}^{\circ}\;C^{-1}\cdot T\left(t\right)+0.506\,\;CU\,\;day^{-1}, & -3.4{}^{\circ}C<T(t)\leq 3.5{}^{\circ}C\\ -0.159\,\;\;CU\,\;day^{-1}{}^{\circ}C^{-1}\cdot T\left(t\right)+1.621\,\;CU\,\;day^{-1}, & 3.5{}^{\circ}C<T\left(t\right)\leq 10.4{}^{\circ}C\\ 0\,\;CU\,\;day^{-1}, & T\left(t\right)>10.4{}^{\circ}C \end{array}\,\;\;\;\;\;\;\;\;\;\;\;\;\;\;\;\;(2)$$

where *M_chl_(t)* is the rate of chilling in chilling units (CU) per day, and *T(t)* is the prevailing air temperature.

**FIGURE 3:**
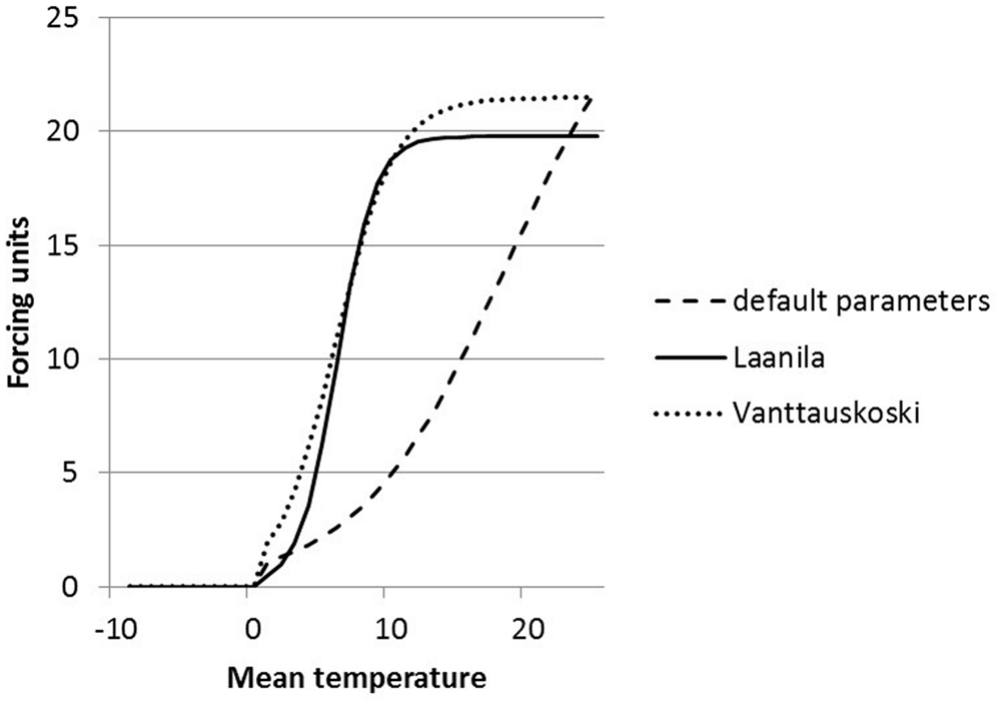
**Forcing unit (FU) equation response to temperature on three different parameter sets; defaults (dashed line), parameters solved for Laanila (solid line), and parameters solved for Vanttauskoski (dotted line)**.

The parameters of the FU equation (Eq. 1) were changed as a result of optimization. The default values (*a* = 28.361, *b* = 0.185, *c* = 18.431) are based on pooled data for several boreal species (Sarvas, 1974) and thus are not species-specific. The parameter values obtained in this study are quite different compared to the default values. Parameter *b* that defines the slope of the temperature response curve received a high value compared to the default value, which resulted in a much steeper curve (**Figure 3**). Parameter *c* defines the shape of the curve, i.e., the temperature at the turning point of the response curve. The parameter values are dependent on each other and the change in the slope has an effect also on the shape.

### BUD-BREAK RECONSTRUCTION

**FIGURE 4:**
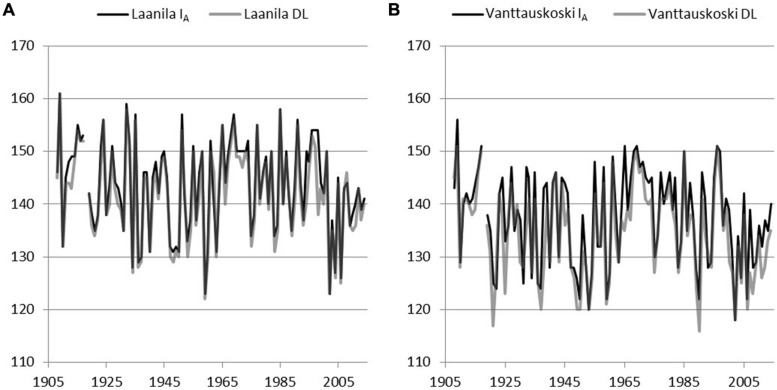
**Reconstructed bud break using adjusted temperature records from Sodankylä and parameters of two models, Model I_**A**_ with a chilling component and Model DL with day-length-triggered forcing, solved for Laanila **(A)** and Vanttauskoski **(B)**.** Year 1918 was omitted due to the missing temperature observations.

**FIGURE 5:**
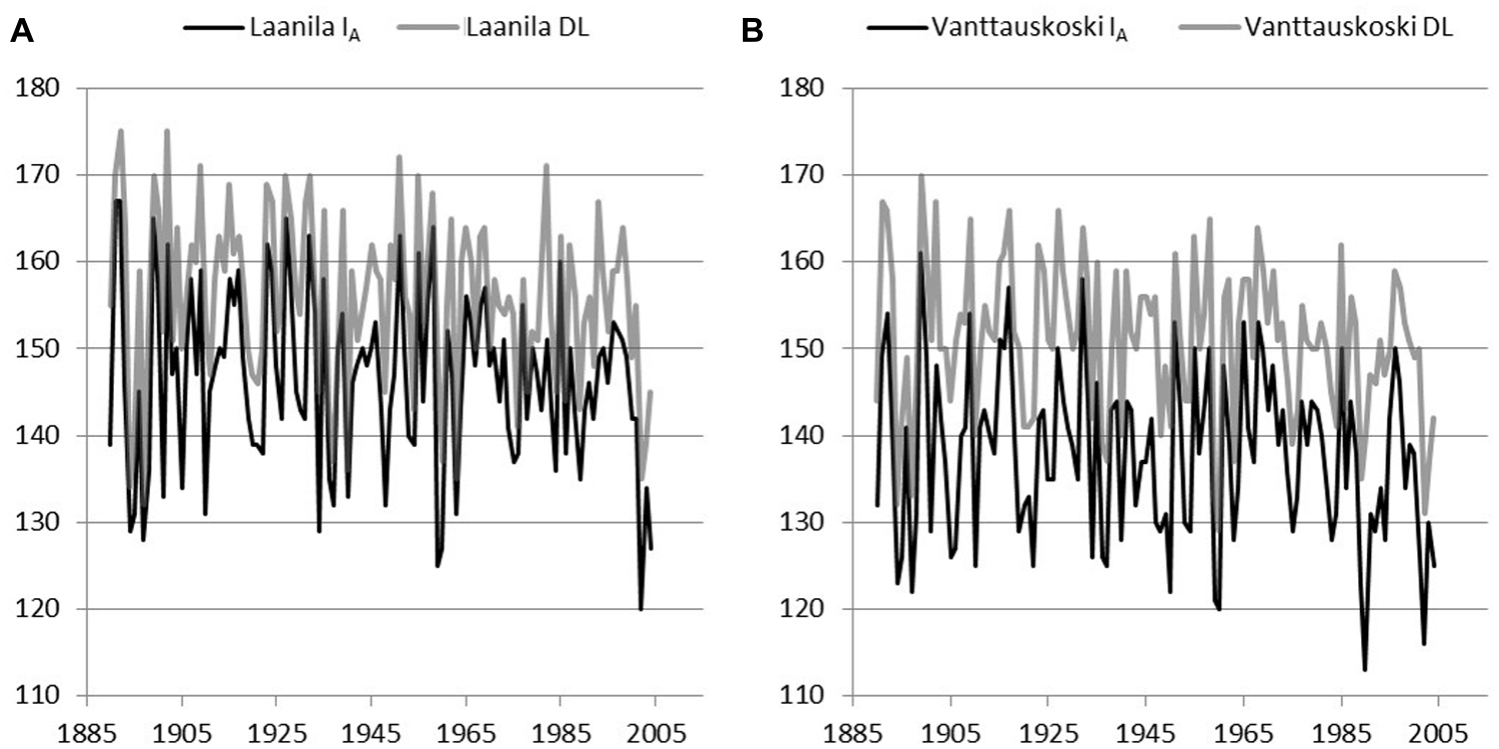
**Reconstructed bud break using adjusted temperature records from Karasjok and parameters of two models, Model I_**A**_ with a chilling component and Model DL with day-length-triggered forcing, solved for Laanila **(A)** and Vanttauskoski **(B)**.** Years 1877–1889 were omitted due to missing temperature observations.

Model I_A_ (includes a chilling effect) and the day-of-year Model DL were used to reconstruct bud-break dates based on the daily temperature record from Sodankylä and Karasjok since the year 1908 and 1877, respectively. The difference between April and May mean temperatures in each of the sites during the model calibration years was used to scale Sodankylä and Karasjok temperature records. Hence, when using Sodankylä data with parameters from Laanila, daily temperature values were reduced by 1.4°C and when using parameters from Vanttauskoski increased by 0.7°C. Likewise, when bud break in Laanila and Vanttauskoski were reconstructed with data from Karasjok, temperature was reduced by 0.4°C and increased by 1.7°C, respectively. Thus, the form of the reconstructed bud-break dates remained intact but their level was in line with the mean temperatures of the calibration data. Due to the missing observations in April–May for temperatures in Sodankylä and Karasjok, the respective years 1918 and 1877–1889 were omitted from the results (**Figures 4** and **5**).

In Laanila, Model I_A_ (includes a chilling-effect) predicted later bud break than the Model DL with temperature data from Sodankylä data, while data from Karasjok the order was reversed (**Table 6**). The difference between the Sodankylä and Karasjok temperature records is strongly affected by the adjustments based on the April–May temperatures. Hence, the absolute values produced based on these two temperature records cannot be directly compared. Instead, the focus should be on trends and variation.

**Table 6:** Mean bud-break dates (day of year) with two temperature records, Sodankylã and Karasjok, and two models (IA and DL) parameterized for Laanila andVanttauskoski.

	Laanila, Model IA	Laanila, Model DL	Vanttauskoski, Model IA	Vanttauskoski, Model DL
Sodankylã	144	142	138	135
Karasjok	146	155	137	150

A time-series analysis revealed that none of the constructed series included statistically significant autocorrelation. A possible linear trend in bud-break dates was analyzed by fitting an autoregressive model to the data. Series based on temperature records from Sodankylä did not include a significant linear trend. Temperature records from Karasjok gave slightly contradictory results. Model I_A_ based on parameters for Vanttauskoski produced a series with no statistically significant trend, but the three other series, Model DL for Vanttauskoski and Models I_A_ and DL for Laanila, included a trend that was statistically significant according to the maximum likelihood (ML) estimates but insignificant based on ordinary least squares (OLS) estimates. The former are known to overestimate residual variance while the latter underestimates it (SAS User’s Guide, 2002). Therefore, the conclusion is that there is a linear trend that is borderline statistical significant (*p* = 0.05). Coefficients for the time-variable in the ML estimation in the Karasjok data ranged from –0.028 to –0.045, which corresponds to a 3–4 day advance in bud break in the last 107 years.

## DISCUSSION

Clark et al. (2014) point out several sources of error in phenological modeling. Our study is not free of them. We set a minimum requirement for measured height growth after bud break was observed and applied linear interpolation when turning weekly observations into a precise date. The weekly measurement interval is a compromise between costs and data precision. Furthermore, the chilling and forcing models were driven by daily mean temperature data, which flattens out the effect of minimum and maximum values, while we did not pay attention to solar radiation, soil temperature, ground frost, or snow melt. These simplifications were due to the relatively short and incomplete data records available.

Comparison of the modeled and observed bud-break dates revealed that due to the definition of bud break, the models performed better when the temperature change from cool to warm was clear and steep. In contrast, when the temperature change was slow, the models gave larger RMSE values. A typical example is seen in 2008 in the southern location, when half of the trees began to grow followed by a break of over a week, after which the rest of the trees also broke. According to the chosen criteria, the time when the latter group of trees started to elongate was selected as the bud-break date. Daily observations and longer continuous field measurements would enable more reliable calibration of the models.

Bud-break models by Hänninen (1990) were calibrated with field observations from two different locations in northern Finland. Empirical material consists of five different tree groups, each followed for three consecutive years. These sample data were fed into an optimization procedure that selected parameters for chilling and forcing models and which gave the lowest RMSE. Both pure numerical parameters and fixed-day-of-year, when applicable, were analyzed simultaneously. Our approach exemplifies how young trees of today would have reacted to the chilling and forcing temperatures of the past climate. The size of the data does not justify extensive generalization and rather than focusing on the parameters of the models, the emphasis is on the trends shown by the reconstructed bud-break series. There are visible cyclic changes but no clear long-term trends that can be distinguished in the series based on Sodankylä temperature data; the bud-break dates for the 1920s and the 1950s are as early as those seen in the beginning of the 2000s. This is not consistent with the results of recent articles in the field of phenology, which have usually confirmed a long-term temperature rise (e.g., Linkosalo et al., 2009). This study is based on observations from northern Finland and focus on early spring temperature, which may explain why findings are not the same when analyzing southern locations and temperature later in the growing season. On the other hand, data from Karasjok indicated that there is a linear trend, although its statistical significance was not very clear. Although observations from Karasjok and Sodankylä are similar to a large degree, the April and May mean temperatures from Karasjok in particular have a small increasing trend over the century that makes a difference. Recent findings, for example, on temperature development based on dendroclimatological studies of pine in the upper Fennoscandia and NW Russia (McCarroll et al., 2013; Lindholm et al., 2014) are in line with our results on thermal development.

Our results of a trend towards earlier bud burst in pine are not entirely in keeping with results for downy birch (*Betula pubescens* Ehrh.) from the same area, which had predicted the potential growing season would increase considerably (Bennie et al., 2010). Similarly Heikinheimo and Lappalainen (1997) and Pudas et al. (2008) see that springs seasons at high latitudes have advanced. The reason for the disparity centers on the length of the time span; put simply, one is unable to suggest spring advance with just data covering 7–15 years. Even given the length of our reconstruction period, the maximum instrumental period since 1908 in Sodankylä may be too short, since it cannot indicate low frequencies in the series at all.

## Conflict of Interest Statement

The authors declare that the research was conducted in the absence of any commercial or financial relationships that could be construed as a potential conflict of interest.
